# UBIAD1 Plays an Essential Role in the Survival of Pancreatic Acinar Cells

**DOI:** 10.3390/ijms20081971

**Published:** 2019-04-22

**Authors:** Kimie Nakagawa, Kiyomi Fujiwara, Akihiro Nishimura, Chinami Murakami, Kanaha Kawamoto, Chihiro Ichinose, Yumi Kunitou, Yoshitomo Suhara, Toshio Okano, Hiroshi Hasegawa

**Affiliations:** 1 Laboratory of Hygienic Sciences, Kobe Pharmaceutical University, Kobe 658-8558, Japan; 194k38change@gmail.com (K.F.); sweet.vanilla0627@gmail.com (A.N.); chi73.smile@icloud.com (C.M.); knha.3mal@gmail.com (K.K.); ap3-m22@docomo.ne.jp (C.I.); ow_kqqhqfqq_5qjn111@docomo.ne.jp (Y.K.); t-okano@kobepharma-u.ac.jp (T.O.); h-hase@kobepharma-u.ac.jp (H.H.); 2 Laboratory of Organic Synthesis and Medicinal Chemistry, College of Systems Engineering and Science, Faculty of Bioscience and Engineering, Shibaura Institute of Technology, Saitama 337-8570, Japan; suhara@shibaura-it.ac.jp

**Keywords:** UBIAD1, MK-4, tamoxifen, knockout mice, pancreas, acinar cells

## Abstract

UbiA prenyltransferase domain-containing protein 1 (UBIAD1) is a vitamin K_2_ biosynthetic enzyme. We previously showed the lethality of this enzyme in UBIAD1 knockout mice during the embryonic stage. However, the biological effects of UBIAD1 deficiency after birth remain unclear. In the present study, we used a tamoxifen-inducible systemic UBIAD1 knockout mouse model to determine the role of UBIAD1 in adult mice. UBIAD1 knockout resulted in the death of the mice within about 60 days of administration of tamoxifen. The pancreas presented with the most prominent abnormality in the tamoxifen-induced UBIAD1 knockout mice. The pancreas was reduced remarkably in size; furthermore, the pancreatic acinar cells disappeared and were replaced by vacuoles. Further analysis revealed that the vacuoles were adipocytes. UBIAD1 deficiency in the pancreatic acinar cells caused an increase in oxidative stress and autophagy, leading to apoptotic cell death in the tamoxifen-induced UBIAD 1 knockout mice. These results indicate that UBIAD1 is essential for maintaining the survival of pancreatic acinar cells in the pancreas.

## 1. Introduction

Vitamin K is a cofactor for γ-glutamyl carboxylase (GGCX), an enzyme that converts specific glutamic acid residues in several substrate proteins involved in blood coagulation and bone metabolism to γ-carboxyglutamic acid residues [1,2]. In addition to its role as a cofactor for GGCX, vitamin K is involved in the transcriptional regulation of the nuclear receptor the steroid and xenobiotic sensing nuclear receptor (SXR) (also known as pregnane X receptor or PXR) [3,4,5] and regulates protein kinase A (PKA) signaling in osteoblasts and hepatocellular carcinoma cells [6]. Furthermore, menaquinone-4 (MK-4) is reported to have a role in the mitochondrial electron transport chain in *Drosophila* [7].

There are two naturally occurring forms of vitamin K, phylloquinone (PK or vitamin K_1_) and the group of menaquinones (MKs). All forms of vitamin K are characterized by a common 2-methyl-1,4-naphthoquinone ring structure (menadione, MD) and a hydrophobic polyisoprenoid side chain attached at the 3-carbon position of the nucleus. PK has a monounsaturated side chain of four isoprenyl residues and is primarily found in leafy green vegetables. MKs can be classified into 14 types on the basis of the length of their unsaturated side chains. Among them, MK-4 is an atypical menaquinone that is not commonly synthesized by bacteria but can be synthesized in vivo by invertebrates and vertebrates in the presence of a suitable naphthoquinone precursor, such as MD or PK [8,9].

We recently demonstrated that dietary PK releases MD into the mesenteric lymphatic system and blood circulation via cleavage of the side chain in the intestine, following which it is delivered to the tissues where it is converted into MK-4 and stored [8]. The UbiA prenyltransferase domain-containing protein 1 (UBIAD1) is a novel MK-4 biosynthetic enzyme screened and identified from the human genome database [10,11]. Enzymatic analysis using site-directed mutagenesis revealed that UBIAD1 is capable of generating various MKs, including MK-4; additionally, UBIAD1 missense mutations were found to significantly lower MK-4 biosynthetic activity in Schnyder corneal dystrophy [12,13]. To clarify the function of UBIAD1 in vivo, we attempted to generate mice completely lacking *Ubiad1*, a homolog of human UBIAD1, by gene targeting. Conventional *Ubiad1*-deficient (*Ubiad1*^−/−^) mouse embryos failed to survive beyond embryonic day 7.5, exhibiting a small-sized body and gastrulation arrest, while the *Ubiad1*^+/−^ mice exhibited normal growth and fertility [14]. The tissue concentrations and synthetic activity of MK-4 in *Ubiad1*^+/−^ mice were approximately half of those in the wild-type mice. The *Ubiad1^−/−^* mouse embryos could not be rescued, but their embryonic lifespans were extended to term by oral administration of MK-4 or CoQ10 in pregnant *Ubiad1*^+/−^ mice [14]. These results suggest that UBIAD1 plays a critical role in embryonic development by synthesizing MK-4.

UBIAD1 is an essential factor for mouse development, but it is not possible to conduct a functional analysis of this protein in each tissue from the fetal stage in UBIAD1-deficient mice. The goal of this study was to determine the role of UBIAD1 in the physiological function in adult mice. In order to determine the role of UBIAD1 in several tissues in adult mice, we created tamoxifen-inducible generalized UBIAD1 knockout mice and identified the novel functions of UBIAD1 in these mice after maturation.

## 2. Results

### 2.1. Tamoxifen-Induced Ubiad1-Deficient Mice Are Lethal

**Figure 1:**
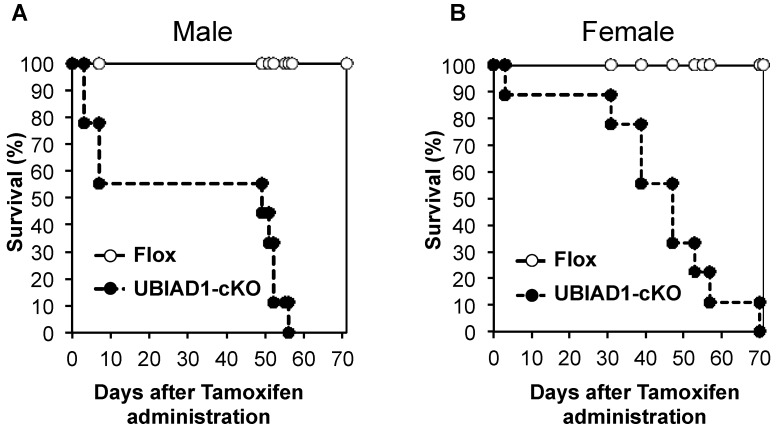
Kaplan–Meier survival curves of male (a) and female (b) Flox and UbiA prenyltransferase domain-containing protein 1 knockout (UBIAD1-cKO) mice after administration of tamoxifen.

CAG-Cre-ERT^+/−^UBIAD1^fl/fl^ (UBIAD1-cKO) mice injected with tamoxifen died within 60–70 days of tamoxifen administration. Tamoxifen-treated UBIAD1-cKO male mice died about 60 days after tamoxifen administration, while the female mice died after 70 days (Figure 1).

### 2.2. The Pancreas of Tamoxifen-Induced UBIAD1-Deficient Mice Was Markedly Atrophied

**Figure 2:**
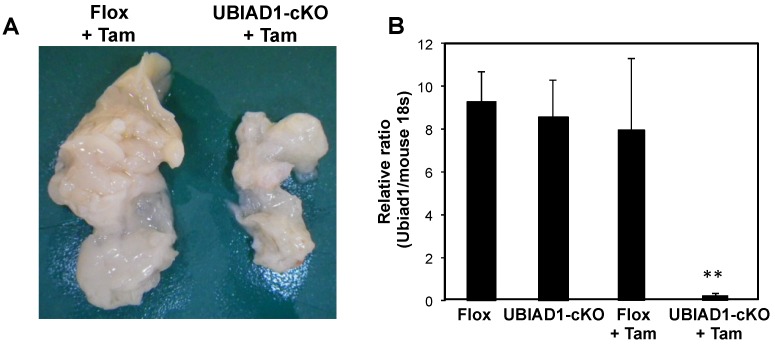
Morphological changes and UBIAD1 messenger RNA (mRNA) expression in the pancreas of tamoxifen-treated UBIAD1-cKO female mice. (**A**) Morphological changes in the pancreas of tamoxifen-treated Flox and UBIAD1-cKO female mice; (**B**) UBIAD1 mRNA expression in the pancreas of tamoxifen-treated or non-treated Flox and UBIAD1-cKO female mice. Data are shown as means ± standard errors of the mean (SEM); ** *p* < 0.01 compared with the corresponding value in other group of mice.

Female UBIAD1-cKO mice were analyzed because they seemed to survive for longer periods than male mice. Body and tissue weights of UBIAD1-cKO female mice were measured at 40 days after administration of tamoxifen and compared with those of the tamoxifen-treated CAG-Cre-ERT^−/−^UBIAD1^fl/fl^ (Flox) mice carrying conditional UBIAD1 alleles, but lacking Cre expression (Table 1). No significant differences in body weights were noted between the Flox and UBIAD1-cKO mice (both tamoxifen-treated and non-treated). However, the weight of the pancreas in the tamoxifen-treated UBIAD1-cKO mice was markedly reduced when compared with the Flox mice and the tamoxifen non-treated UBIAD1-cKO mice (Table 1). On the other hand, the weights of other tissues were not significantly different among the four types of mice (Table 1). MK-4 and deuterium-labeled MK-4 (MK-4-d_7_) concentrations converted from deuterium-labeled MD (MD-d_8_) in the tamoxifen-treated UBIAD1-cKO mice were significantly lower than in the tamoxifen-treated Flox mice (Table 2). Surprisingly, the pancreas of the tamoxifen-treated UBIAD1-cKO mice was markedly atrophied when compared with the tamoxifen-treated Flox mice, as shown in Figure 2A. Furthermore, UBIAD1 messenger RNA (mRNA) expression levels were markedly reduced in the tamoxifen-treated UBIAD1-cKO mice when compared with the tamoxifen-treated Flox mice and the tamoxifen non-treated UBIAD1-cKO mice (Figure 2B). UBIAD1 mRNA levels in other tissues were also markedly reduced in the tamoxifen-treated UBIAD1-cKO mice (Figure S2, Supplementary Materials).

### 2.3. Disappearance of Pancreatic Acinar Cells and Formation of Vacuoles in the Pancreas of Tamoxifen-Induced UBIAD1-Deficient Mice

**Figure 3:**
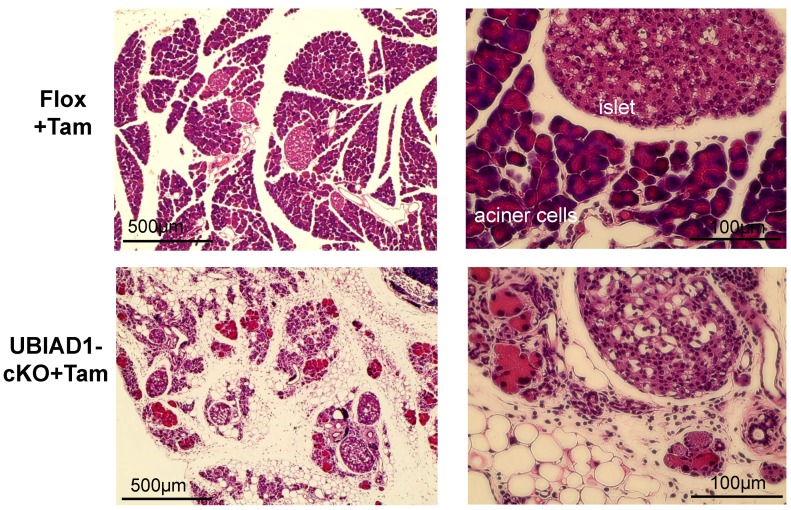
Hematoxylin–eosin (H&E)-stained pancreas sections from female Flox and UBIAD1-cKO mice at 40 days after administration of tamoxifen.

**Figure 4:**
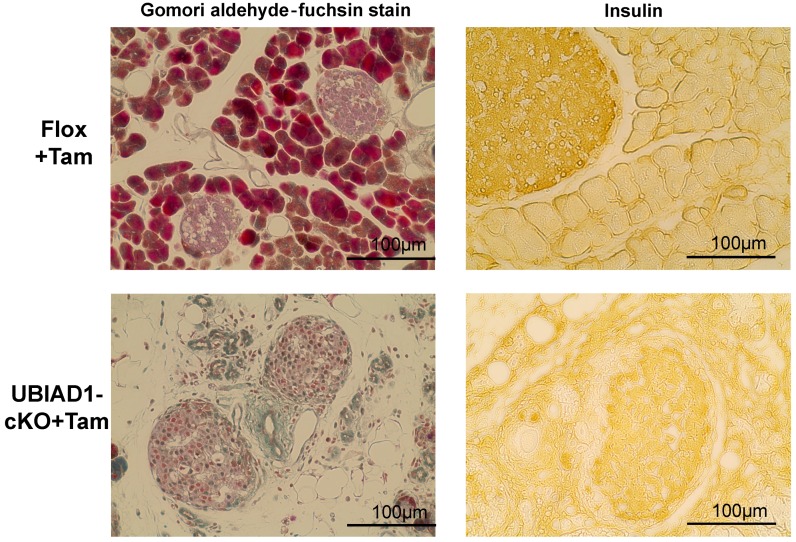
Gomori aldehyde fuchsin and insulin immune-stained pancreas sections from female Flox and UBIAD1-cKO mice at 40 days after administration of tamoxifen. In the Gomori aldehyde fuchsin-stained sections, elastic fibers and pancreatic islet β class cells were stained purple, pancreatic islet δ cells were stained light green, pancreatic islet α cells were stained red, and connective tissue was stained green. Immunohistological staining with insulin specific antibody in the pancreas of the tamoxifen-treated Flox and UBIAD1-cKO mice at 40 days after administration of tamoxifen.

The pancreas of the tamoxifen-treated UBIAD1-cKO mice was remarkably smaller. Tissue sections were prepared, and hematoxylin–eosin (H&E) staining was performed. Pancreatic acinar cells were absent in the tamoxifen-treated UBIAD1-cKO mice; instead, several vacuoles were noted (Figure 3). On the other hand, the presence of internal α, β, and δ cells was confirmed by Gomori aldehyde fuchsin staining (Figure 4). No atrophy of the pancreatic islets of tamoxifen-treated UBIAD1-cKO mice was noted. The pancreas of tamoxifen-treated Flox mice was stained with insulin antibody only in the pancreatic islets, whereas, in tamoxifen-treated UBIAD1-cKO mice, pancreatic islets and portions other than the pancreatic islets were also widely stained (Figure 4). These results indicate that insulin secreted from pancreatic islets is dispersed within the pancreas in tamoxifen-treated UBIAD1-cKO mice.

### 2.4. The Vacuoles Found in the Pancreas of Tamoxifen-Treated UBIAD1-cKO Mice Were Adipocytes

**Figure 5:**
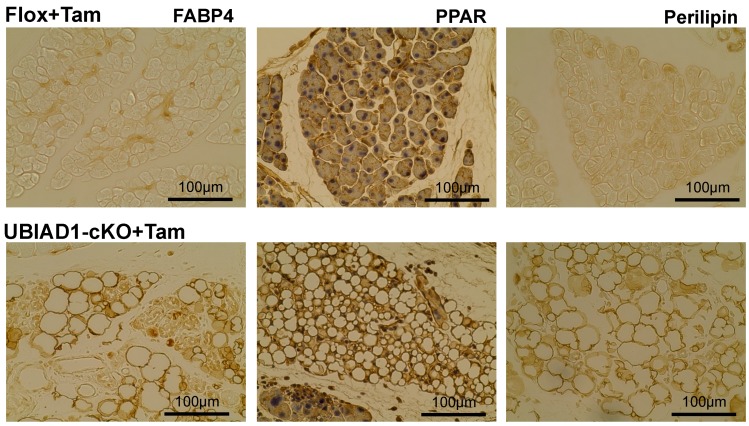
Expression of adipocyte-specific markers in the vacuoles in tamoxifen-treated UBIAD1-cKO mice. Immunohistological staining with FABP4, PPAR, and perilipin in the pancreas of the tamoxifen-treated Flox and UBIAD1-cKO mice at 40 days after administration of tamoxifen. The tissues of immunohistological staining with PPAR staining were counterstained with hematoxylin.

Immunohistochemical staining using adipocyte-specific antibodies (Fatty acid binding protein 4 (FABP4), Peroxisome proliferator-activated receptor (PPAR), and perilipin) was performed to investigate the vacuoles found in the pancreas of the tamoxifen-treated UBIAD1-cKO mice (Figure 5). These results indicate that, in the pancreas of tamoxifen-treated UBIAD1-cKO mice, pancreatic acinar cells disappeared and adipocytes were formed at the disappeared site.

### 2.5. Pancreatic Acinar Cells in the Pancreas of Tamoxifen-Treated UBIAD1-cKO Mice Increased Oxidative Stress and Autophagy and Disappeared by Apoptosis

**Figure 6:**
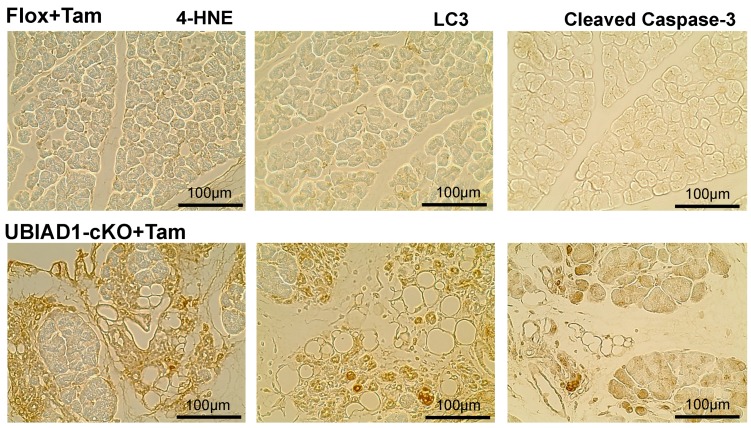
Expression of oxidative stress, autophagy, and apoptosis-specific markers 4-HNE, LC3, and cleaved caspase-3, respectively, in the pancreas of tamoxifen-treated Flox and UBIAD1-cKO mice at 40 days after administration of tamoxifen.

In order to determine the cause of the formation of vacuoles and the disappearance of pancreatic acinar cells in the cKO mice, we investigated the involvement of oxidative stress, autophagy, and apoptosis. Strong 4-Hydroxy nonenal (4-HNE)-positive staining observed at the site where the acinar cells disappeared indicated that the pancreatic acinar cells in tamoxifen-treated UBIAD1-cKO mice were exposed to strong oxidative stress. In addition, positive staining for Microtubule-associated protein light chain 3 (LC3), a central protein in the autophagy pathway, was detected in atrophied pancreatic acinar cells in the tamoxifen-treated UBIAD1-cKO mice. Furthermore, cleaved caspase-3 positive cells were observed in the atrophic and non-atrophic pancreatic acinar cells (Figure 6). These findings indicated that pancreatic acinar cells in the tamoxifen-treated UBIAD1-cKO mice disappeared via apoptosis due to oxidative stress and enhancement of autophagy.

### 2.6. Abundance of Neutrophils and Mesenchymal Stem Cells in the Pancreas of Tamoxifen-Treated UBIAD1-cKO Mice

**Figure 7:**
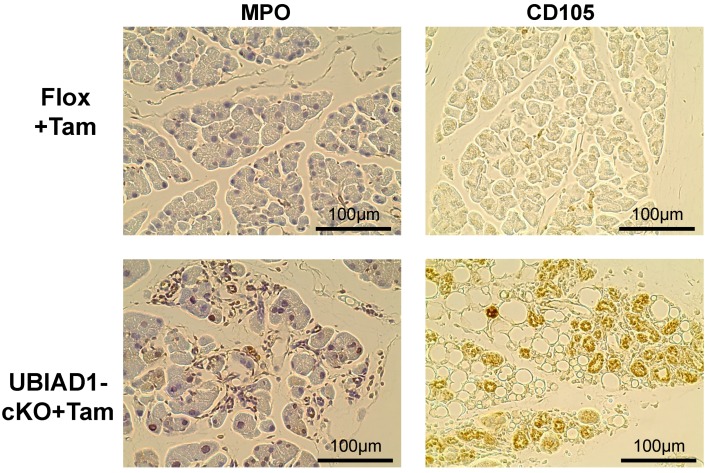
The pancreas of the tamoxifen-treated UBIAD1-cKO mice was positive for the inflammation (myeloperoxidase; MPO) and mesenchymal stromal cell (CD105) markers. Immunohistological staining of MPO and CD105 in the pancreas of tamoxifen-treated Flox and UBIAD1-cKO mice at 40 days after administration of tamoxifen. The tissues of immunohistological staining with MPO staining were counterstained with hematoxylin.

The pathology of the pancreas in tamoxifen-treated UBIAD1-cKO mice is similar to that of chronic pancreatitis. Several cells positively stained for myeloperoxidase (MPO), a neutrophil marker, were detected in the pancreas of tamoxifen-treated UBIAD1-cKO mice (Figure 7). Furthermore, in order to investigate whether undifferentiated mesenchymal stem cells were differentiated into adipocytes in the pancreatic tissue, the cells were stained with an antibody against CD105 (as known as Endoglin), a mesenchymal stem cell marker. As a result, several CD105-positive cells were detected in the pancreas of the tamoxifen-treated UBIAD1-cKO mice (Figure 7). These results suggest that the mesenchymal stem cells may have invaded during inflammation and differentiated into adipocytes in the pancreas of these mice.

### 2.7. Serum Glucose and Insulin Concentrations and Glucose Tolerance in Tamoxifen-Induced UBIAD1-Deficient Mice

**Figure 8:**
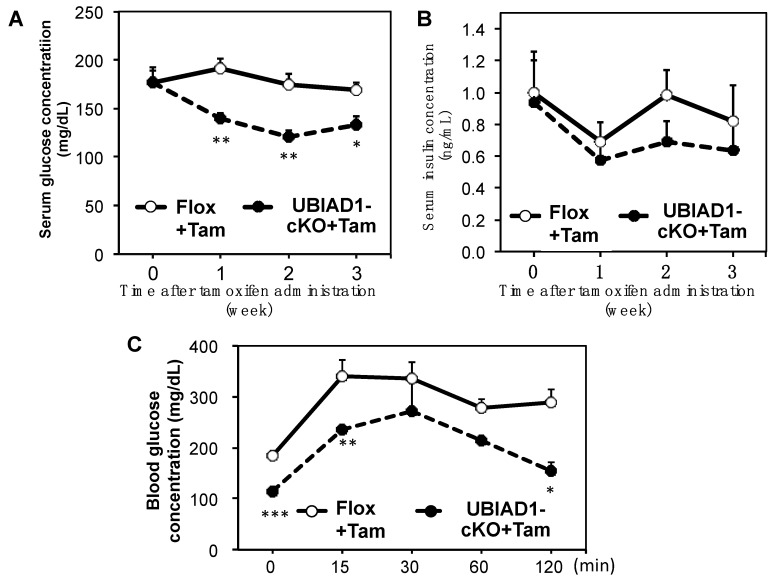
Serum glucose (**A**) and insulin (**B**) concentrations in Flox and UBIAD1-cKO mice after tamoxifen administration. Oral glucose tolerance test (**C**) in tamoxifen-treated Flox and UBIAD1-cKO mice at 40 days after administration of tamoxifen. Serum glucose was assayed at 0, 15, 30, 60, and 120 min. Each point on the curve represents the mean ± SEM. Data are shown as means ± SEM; * *p* < 0.05 and ** *p* < 0.01 when compared with the corresponding values at each point in the tamoxifen-treated Flox mice.

We measured the serum glucose and insulin concentrations in the Flox and UBIAD1-cKO mice every week following tamoxifen administration. Serum glucose concentration was significantly lowered in tamoxifen-treated UBIAD1-cKO mice when compared with the tamoxifen-treated Flox mice; likewise, the concentration of serum insulin in these mice tended to be lower than that in the Flox mice (Figure 8A,B). Therefore, we conducted a glucose tolerance test in the two types of mice 40 days after tamoxifen administration. The blood glucose level in the tamoxifen-treated UBIAD1-cKO mice at 0 min before glucose loading was significantly lower than that of the tamoxifen-treated Flox mice. A slight increase in the level was noted in the tamoxifen-treated UBIAD1-cKO mice after glucose loading; however, they remained significantly lower in these mice when compared with the tamoxifen-treated Flox mice (Figure 8C) indicating that tamoxifen-induced UBIAD1 deficient mice may have decreased glucose absorption capacity and pancreatic function.

### 2.8. Abnormal Tissue Structure and Decreased Sodium-Dependent Glucose Transporter 1 (SGLT1) and Glucose Transporter 2 (GLUT2) Expressions in the Duodenum of Tamoxifen-Treated UBIAD1-cKO Mice

**Figure 9:**
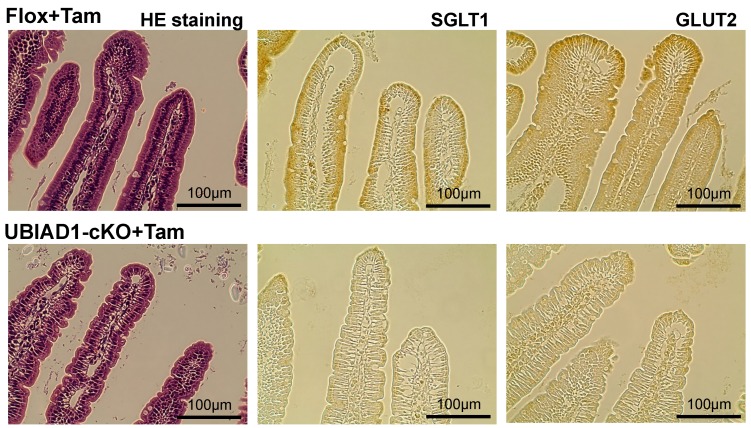
H&E-stained and immunohistologically stained duodenum sections from female Flox and UBIAD1-cKO mice at 40 days after administration of tamoxifen. Immunohistological staining demonstrated sodium-dependent glucose transporter 1 (SGLT1) and glucose transporter 2 (GLUT2) expressions in the duodenum of the mice at 40 days after tamoxifen administration

UBIAD1 is deficient not only in the pancreas but also in the duodenum of the tamoxifen-treated UBIAD1-cKO mice, thereby affecting the glucose absorption capacity of the duodenum. The basolateral membrane in the duodenum of the tamoxifen-treated UBIAD1-cKO mice appeared disorganized, while the surface of intestinal villus was rough when compared with those in the tamoxifen-treated Flox mice (Figure 9). Glucose is absorbed through the intestine by a transepithelial transport system that is initiated at the apical membrane by the co-transporter SGLT1; intracellular glucose is then assumed to diffuse across the basolateral membrane through GLUT2. Marked SGLT1 and GLUT2 expressions were observed on the luminal side of the villi in the duodenum of the tamoxifen-treated Flox mice, but not in the tamoxifen-treated UBIAD1-cKO mice (Figure 9). Expression of SGLT1 and GLUT2 was very weak in duodenal epithelial cells of the tamoxifen-treated UBIAD1-cKO mice.

## 3. Discussion

UBIAD1 is an essential enzyme required for MK-4 biosynthesis in mice and humans. The conventional UBIAD1 knockout mice do not survive during the early gestation period, thus indicating that UBIAD1 plays an essential role in the developmental process [14]. UBIAD1 exists in all tissues, and is expected to maintain the function of each tissue by biosynthesizing MK-4; nonetheless, the significance of this biosynthesis in each organization remains unclear. Therefore, in order to clarify the importance and role of UBIAD1 in each tissue in mice after birth, we generated a tamoxifen-induced systemic UBIAD1-deficient mice model and evaluated the effects of UBIAD1 deficiency in these mice after maturation.

In the present study, the UBIAD1-deficient mice did not survive after maturation. Female and male UBIAD1-cKO mice demonstrated similar rates of death after administration of tamoxifen, but male UBIAD1-cKO mice died earlier than the female mice. Previously, we reported that the concentration of MK-4 in tissues of male wild-type mice was lower than that of the female wild-type mice [9]. Hence, it was considered that male UBIAD1-cKO mice in which UBIAD1 deficiency was induced by tamoxifen administration became MK-4-deficient earlier than female UBIAD1-cKO mice, leading to lethality. The body and tissue weights of female UBIAD1-cKO mice were measured 40 days after induction of UBIAD1 deficiency by tamoxifen. No significant differences in body weight were noted between the UBIAD1-cKO and Flox mice treated with tamoxifen, whereas the weight of the pancreas in the UBIAD1-cKO mice was significantly lower than that in the Flox mice. The pancreas of tamoxifen-treated UBIAD1-cKO mice was markedly atrophied and reduced in size; additionally, MK-4 concentration and UBIAD1 expression were decreased in the whole tissues of these mice. It is known that the concentration of MK-4 is highest in the pancreas when compared with any other tissue [9]. In a previous study, Thomas et al. indicated that MK-4 is detected in pancreatic secretions and is present within the secretory pathway of acinar cells, thereby suggesting that MK-4 has a unique function in acinar cells [15]. Consistent with the low concentrations of phylloquinone reported in exocrine tissues [16,17], no vitamin K compounds other than MK-4 were detected in pancreatic secretions. Thomas et al. reported that the detection of MK-4 in pancreatic secretions indicates its presence in the secretory pathway of the acinar cells [15]. Although the reason for the abundance of MK-4 in the pancreas is not clear, the findings of the current study suggest that it may play an important role in this organ.

H&E staining revealed an absence of pancreatic acinar cells and an increase in the number of vacuoles in the tissues of the tamoxifen-treated UBIAD1-cKO mice. The number of pancreatic islets was similar in the tamoxifen-treated UBIAD1-cKO mice and Flox mice, indicating that the abnormality was limited to the pancreatic acinar cells. The pancreas of the tamoxifen-treated UBIAD1-cKO mice was remarkably reduced in size. However, no atrophy of the pancreatic islets of tamoxifen-treated UBIAD1-cKO mice was noted. We considered that the cause of the atrophy of the pancreas of UBIAD1-cKO mice was the loss of pancreatic acinar cells that occupy 90% of the pancreas. Although various digestive enzymes are present in pancreatic acinar cells, it is speculated that UBIAD1 deficiency did not cause significant cell damage in pancreas islets because pancreatic islets do not produce the digestive enzymes. As a result of observing the expression of insulin by immunohistological staining, the insulin in pancreatic islets was stained equally in both tamoxifen-treated Flox mice and UBIAD1-cKO mice. From this, it is speculated that the level of insulin secretion in pancreatic islets is not reduced in cKO mice. However, no insulin was observed in the pancreatic acinar cells of the Flox mice, whereas, in the UBIAD1-cKO mice, extensive insulin diffusion to the site of the pancreatic acinar cells was noted. The pancreatic acinar cells in the tamoxifen-treated UBIAD1-cKO mice almost disappeared, and most of the cells were replaced by vacuoles. Interestingly, the vacuoles stained positively for the adipocyte marker proteins FABP4, PPAR [18,19], and perilipin [20,21], indicating that the pancreatic acinar cells were replaced by fat cells. Although the functions of UBIAD1 and MK-4 in the pancreas are not clear, periostin, a member of a vitamin K-dependent γ-carboxylated protein family, is reported to be involved in pancreatitis [22]. In normal mice, periostin expression is strongly induced throughout the fibrotic reaction in cerulein-mediated acute pancreatitis [23]. However, in periostin-deficient mice, the most striking phenotype of periostin-knockout mice is the replacement of acinar cells by adipocytes after induction of acute pancreatitis [23]. The involvement of periostin was not further examined in the current study. However, MK-4 deficiency is caused by UBIAD1 deficiency in tamoxifen-induced UBIAD1-cKO mice, and since periostin is not γ-carboxylated by GGCX, it is speculated that the acinar cells may have disappeared and been replaced with adipocytes.

In humans and mice, it is reported that vacuoles are formed at the site of pancreatic acinar cells during acute pancreatitis [24]. Pancreatic acinar cells are the functional units of the exocrine pancreas. They are known to synthesize, store, and secrete digestive enzymes. Under normal physiological conditions, digestive enzymes are activated only after they reach the duodenum [25]. Premature activation of these enzymes within pancreatic acinar cells leads to the onset of acute pancreatitis [25], wherein the acinar cells are digested by the enzymes. The causes of acute pancreatitis include oxidative stress and increased autophagy. Oxidative stress and its constant companion, inflammation, play critical roles in the pathogenesis of pancreatitis and its numerous complications [26,27,28,29]. Oxidative stress is caused by a combination of the increased production of reactive oxygen species and impaired antioxidant capacity. Previously, it was reported that autophagy is induced by the supramaximal stimulation of cerulein, and is directly related to trypsinogen activation and the onset of acute pancreatitis in mice [30]. Therefore, we investigated the involvement of oxidative stress, autophagy, and apoptosis in pancreatic acinar cell loss in these mice. The atrophied pancreatic acinar cells in the tamoxifen-treated UBIAD1-cKO mice were positive for the oxidative stress marker 4-HNE [31], and a large amount of LC3 deposition (indicating autophagy) was detected. Furthermore, the pancreatic acinar cells in the tamoxifen-treated UBIAD1-cKO mice were positive for the cleaved caspase-3 apoptotic marker. These results suggested that the pancreatic acinar cells disappeared via apoptosis due to enhanced oxidative stress and autophagy. Previous reports suggested that MK-4 prevents oxidative cell death in developing oligodendrocytes and neurons [32,33], and that UBIAD1 prevents oxidative damage in cardiovascular tissues [34], and sustains mitochondrial function in *Drosophila* [7]. Since both MK-4 and UBIAD1 are thought to be involved in anti-oxidation, the deficiency of both UBIAD1 and MK-4 may result in oxidative stress, autophagy enhancement, and apoptosis in pancreatic acinar cells. Mesenchymal stem cells were shown to differentiate into adipocytes [35]. However, according to the present study, most of the pancreatic acinar cells disappeared probably via apoptosis due to oxidative stress and autophagy; moreover, CD105-positive mesenchymal stem cells were frequently found at the site of these cells. Taken together, these results indicate that, after the disappearance of the pancreatic acinar cells in the tamoxifen-treated UBIAD1-cKO mice, mesenchymal stem cells may have invaded the tissues and differentiated into adipocytes due to stimulation via insulin in the pancreas. Thus, UBIAD1 and MK-4 may play essential roles in the survival and function of pancreatic acinar cells.

Serum glucose concentration was significantly lower in the UBIAD1-cKO mice than in the Flox mice after tamoxifen administration, and both before and after the oral glucose tolerance test (OGTT). This may be due to a decrease in pancreatic function. However, analysis of the duodenal tissue revealed a disordered basolateral membrane in the tamoxifen-treated UBIAD1-cKO mice, and the surface of intestinal villus was rough. Moreover, SGLT1 and GLUT2 expression levels were decreased in the duodenum of the tamoxifen-treated UBIAD1-cKO mice. In the duodenum of tamoxifen-treated UBIAD1-cKO mice, UBIAD1 deficiency is thought to cause abnormalities in intestinal epithelial cell differentiation and maturation. As a result, expression of SGLT1 and GLUT2 in intestinal epithelial cells may be reduced. Intestinal glucose absorption is mediated by SGLT1, but GLUT2 is thought to provide a basolateral outlet. GLUT2 can be recruited to the apical membrane after application of a high-glucose bolus into the lumen to allow for the bulk absorption of glucose via facilitated diffusion. Furthermore, SGLT1 and GLUT2 are suggested to play important roles in intestinal glucose sensing [36,37,38,39]. The decrease in the expression levels of SGLT1 and GLUT2 is the main factor responsible for the reduction in glucose absorption from the intestine in UBIAD1-deficient mice. Thus, UBIAD1 is thought to be necessary for cell proliferation and differentiation, and for maintenance of the function of the intestinal epithelial cells. Previous reports demonstrated a relationship between vitamin K intake and insulin sensitivity or glucose tolerance [40]. However, the action of MK-4 on intestinal glucose absorption remains to be shown. The results of the current study suggest that UBIAD1 and MK-4 play important roles in maintaining the morphology and function of intestinal epithelial cells and the expression levels of SGLT1 and GLUT2, which are important for glucose absorption. Further detailed analyses are required to examine this phenomenon in the future.

In summary, our data demonstrate that the loss of UBIAD1 in adult mice results in decreased survival, particularly due to severe abnormalities in the pancreas. The pathology of the UBIAD1-deficient pancreas is similar to that of acute pancreatitis, resulting in the disappearance of pancreatic acinar cells, which are replaced by adipocytes. The pancreatic acinar cells in UBIAD1-deficient mice demonstrated increased oxidative stress, autophagy, and apoptosis. Furthermore, mesenchymal stem cells in the pancreas may have been exposed to pancreatic insulin and differentiated into adipocytes. These findings indicate that both UBIAD1 and MK-4 play essential roles in maintaining the survival of pancreatic acinar cells.

## 4. Materials and Methods

### 4.1. Materials

MD-d_8_ was purchased from C/D/N Isotopes, Inc. PK epoxide, MK-4 epoxide, ^18^O-labeled PK and MK-4 (PK-^18^O and MK-4-^18^O), deuterium-labeled PK and PK epoxide (PK-d_7_ and PK-d_7_ epoxide), and MK-4-d_7_ and MK-4 epoxide (MK-4-d_7_ epoxide) were synthesized in our laboratory as reported previously [8,21].

### 4.2. Ethics Statement

All animal experimental protocols were performed in accordance with the Guidelines for Animal Experiments at Kobe Pharmaceutical University and were approved by Kobe Pharmaceutical University Committee for Animal Care and Use, Kobe, Japan (2016-001; 1 April 2016, 2017-006; 1 April 2017, 2018-055; 1 April 2018). All surgical procedures were performed under isoflurane anesthesia, and all efforts were made to minimize suffering.

### 4.3. Mice

The UBIAD1-floxed mice were generated, as previously described [14]. These offspring were then backcrossed to C57BL6/J for seven generations. UBIAD1^flox/+^ mice were intercrossed to generate UBIAD1^flox/flox^ mice containing homozygous recombinant alleles. CAG-Cre-ERT2 mice were obtained from Jackson Laboratories (#004453). CAG-Cre-ERT^+/−^UBIAD1^fl/+^ mice were generated by mating Ubiad1^flox/flox^ mice with CAG-Cre-ERT2 mice. CAG-Cre-ERT^+/−^UBIAD1^fl/fl^ (UBIAD1-cKO) mice were generated by mating Ubiad1^flox/flox^ (Flox) mice with CAG-Cre-ERT^+/−^UBIAD1^fl/+^ mice.

### 4.4. Genotyping

Genotypes of the flox allele were confirmed by PCR with a sense primer (Ubiad1-GN forward primer (FP-2), 5′–ATCTCACGAAGCTTGAGTTGGC–3′) and an antisense primer (Ubiad1-GN reverse primer (RP-2), 5′–CCACAGTAAGAGATCTTAAAGGCTA–3′). Genotypes of Cre recombinase allele and inter positive allele (IL2 gene) were confirmed by PCR using the following primers: No.4 Cre-F (5′–AATGCTTCTGTCCGTTTGCC–3′), No.4 Cre-R (5′–CTACACCAGAGACGGAAATC–3′), InterPosi (IL2-42)-F (5′–CTAGGCCACAGAATTGAAAGATCT–3′), and InterPosi (IL2-43)-R (5′–GTAGGTGGAAATTCTAGCATCATCC–3′). PCR was performed using 35 cycles with denaturation at 95 °C for 15 s, annealing at 62 °C for 30 s, and extension at 72 °C for 1 min. The predicted PCR products were Flox allele (1.6 kbp), wild-type allele (1.4 kbp), Cre (562 bp), and InterPosi (324 bp) (Figure S1, Supplementary Materials).

### 4.5. Real-Time PCR

Total RNA was isolated from mouse tissue using Isogen (Nippon Gene, Tokyo, Japan) according to the manufacturer’s protocol. First-strand complementary DNA (cDNA) synthesis was performed using ReverTra Ace qPCR RT Master Mix with genomic DNA (gDNA) Remover (TOYOBO, Tokyo, Japan). The cDNA was mixed with SsoAdvanced Universal SYBR Green Supermix (Bio-Rad, Hercules, CA, USA), and amplified using the CFX96 real-time PCR system (Bio-Rad). Mouse *Ubiad1* (GenBank NM_027873, FP: 1575–1595, RP: 1754–1774) and mouse *18s-ribosomal RNA* (GenBank AK135936, FP: 438–455, RP: 486–505) were used in this study.

### 4.6. Measurements of MK-4 and MK-4-d_7_ in Tissues of Mice Administered MD-d_8_

The mice were administered MD-d_8_ orally as a single dose (10 μmol/kg body weight). After 24 h, the animals were sacrificed, and tissues were removed and stored at −80 °C for analysis. MK-4, MK-4 epoxide, MK-4-d_7_, and MK-4-d_7_ epoxide levels were measured by LC-APCI-MS/MS as reported previously [8,21].

### 4.7. Histology and Immunohistochemistry

For histological analysis, tissues were fixed in 4% paraformaldehyde and phosphate-buffered saline (PBS) at 4 °C for 20 h and embedded in a paraffin block. The sections were stained with H&E and Gomori’s aldehyde fuchsin staining (Muto Pure Chemicals Co. Ltd., Tokyo, Japan) according to the manufacturer’s recommendations. The following polyclonal antibodies were used: anti-FABP4 (ab92501, Abcam, Cambridge, UK), anti-PPAR gamma (ab209350, Abcam), anti-perilipin A (ab3526, Abcam), anti-4-hydroxynonenal (ab46545, Abcam), anti-LC3 (PM036, Medical & Biological Lab., Nagoya, Japan), anti-MPO (ab9535, Abcam), anti-CD105 (ab135528, Abcam), anti-SGLT1 (ab14685, Abcam), and anti-GLUT2 (NBP2-22218, Novus Biologicals, Inc., Centennial, CO, USA). Formalin-fixed, paraffin-embedded mice tissues were deparaffinized and incubated in 3% hydrogen peroxide/PBS for 30 min to quench endogenous peroxidases. The sections were rinsed in PBS and immunostained with each specific antibody diluted in 0.5% PBS/ovalbumin (1:100 or 1:200) at 4 °C overnight after antigen retrieval with HistoVT One buffer (Nacalai Tesque, Tokyo, Japan) at 95 °C for 20 min. The sections were then incubated with the secondary antibody goat anti-rabbit IgG-horseradish peroxidase (HRP) sc-2030 (Santa Cruz Biotechnology, Dallas, TX 75220, USA) diluted in 0.5% PBS/ovalbumin (1:200), for 30 min at room temperature. After incubation with Elite ABC Kit (Vector Laboratories, Burlingame, CA, USA) for 30 min and rinsing with PBS, the tissues were stained with 3,3′-diaminobenzidine (DAB) (Vector Laboratories) for 2 min.

### 4.8. Blood Glucose and Insulin

Glucose Pilot Blood Glucose Monitoring System (Aventiir Biotech, LLC, Carlsbad, CA, USA) was used for measuring blood glucose concentrations in the UBIAD1-cKO and Flox mice before and after tamoxifen administration, according to the manufacturer’s recommendations. Blood was obtained from a tail cut (by removing the distal 2 mm of the tail) and assessed for baseline glucose levels. The remaining blood was processed to plasma and later used to determine the fasting insulin levels. All of the plasma samples were frozen after collection and assayed using the Mouse/Rat Insulin ELISA Kit (Morinaga Institute of Biological Science, Tokyo, Japan) according to the manufacturer’s recommendations.

### 4.9. OGTT

OGTT was performed after 4 h of fasting by oral administration of glucose (2 g/kg body weight). Blood was obtained from a tail cut and assessed for baseline glucose levels using the Glucose Pilot Blood Glucose Monitoring System. The remaining blood was processed to plasma and later used to determine the fasting insulin levels. Subsequently, the mice received 2 g/kg body weight of a 100 mg/mL glucose solution in sterile water delivered by oral gavage. At 15, 30, 60, and 120 min after the administration of glucose, dried blood and tissue were quickly removed from the tail wound, and blood was collected again to measure the glucose concentration and to prepare the plasma samples for measuring insulin levels. All plasma samples were frozen after collection for further analysis.

### 4.10. Statistical Analysis

Data are expressed as means ± standard error of mean. Differences between the mean values were analyzed using the unpaired Student’s *t*-test or Dunnett’s test. A *p*-value <0.05 was considered significant.

## Figures and Tables

**Table 1:** Body and tissue weights in the tamoxifen-treated and untreated Flox and UbiA prenyltransferase domain-containing protein 1 knockout (UBIAD1-cKO) mice. Tam—tamoxifen.

	Flox Mice	Flox Mice + Tam	UBIAD1-cKO	UBIAD1-Cko + Tam
Body weight (g)	23.25 ± 0.39	23.45 ± 0.65	22.54 ± 0.39	22.03 ± 0.46
Cerebrum (g)	0.305 ± 0.023	0.287 ± 0.003	0.311 ± 0.004	0.294 ± 0.003
Cerebellum (g)	0.056 ± 0.005	0.059 ± 0.001	0.057 ± 0.004	0.055 ± 0.002
Liver (g)	0.887 ± 0.041	0.883 ± 0.014	0.872 ± 0.045	0.867 ± 0.020
Kidney (g)	0.257 ± 0.010	0.245 ± 0.006	0.254 ± 0.007	0.246 ± 0.010
Pancreas (g)	0.196 ± 0.011	0.192 ± 0.007	0.192 ± 0.005	0.054 ± 0.011 ***

*** *p* < 0.001 compared to corresponding value in Flox mice, Flox mice + Tam, and UBIAD1-cKO mice.

**Table 2:** Menaquinone-4 (MK-4) and deuterium-labeled MK-4 (MK-4-d_7_) concentrations in the Flox and UBIAD1-cKO mice at 40 days after administration of tamoxifen.

	MK-4 concentration (pmol/g)		MK-4-d_7_ concentration (pmol/g)	
	Flox + Tam	UBIAD1-cKO + Tam	Flox + Tam	UBIAD1-cKO + Tam
Cerebrum	429.67 ± 16.55	135.92 ± 14.67***	20.48 ± 1.15	2.38 ± 0.49***
Cerebellum	801.12 ± 76.35	223.46 ± 26.38**	31.82 ± 5.95	2.67 ± 1.29***
Kidney	232.00 ± 12.03	24.45 ± 2.34***	108.18 ± 14.11	7.18 ± 2.46**
Pancreas	1140.91 ± 108.15	96.24 ± 24.15**	61.69 ± 5.21	N.D.^1^

^1^N.D.: not detected; ** *p* < 0.05 and *** *p* < 0.001 compared to corresponding value in Flox + Tam mice.

## Supplementary Materials

Supplementary materials can be found at https://www.mdpi.com/1422-0067/20/8/1971/s1.

## Abbreviations

4-HNE	4-hydroxynonenal
ES	embryonic stem
FRT	flippase
FRT	flippase recombinant target
GLUT2	glucose transporter 2
MK-4	menaquinone-4
MPO	myeloperoxidase
OGTT	oral glucose tolerance test
PKA	protein kinase A
SEM	standard error of mean
SGLT1	sodium-dependent glucose transporter 1
UBIAD1	UbiA prenyltransferase domain-containing protein 1
