# Supplementary material for: UBIAD1 Plays an Essential Role in the Survival of Pancreatic Acinar Cells
Source: Int J Mol Sci. 2019 Apr 22;20(8):1971. doi: 10.3390/ijms20081971 (PMC6515134; doi:10.3390/ijms20081971)
Supplement: Supplementary file 1 [file ijms-20-01971-s001.pdf]

Supplementary Materials

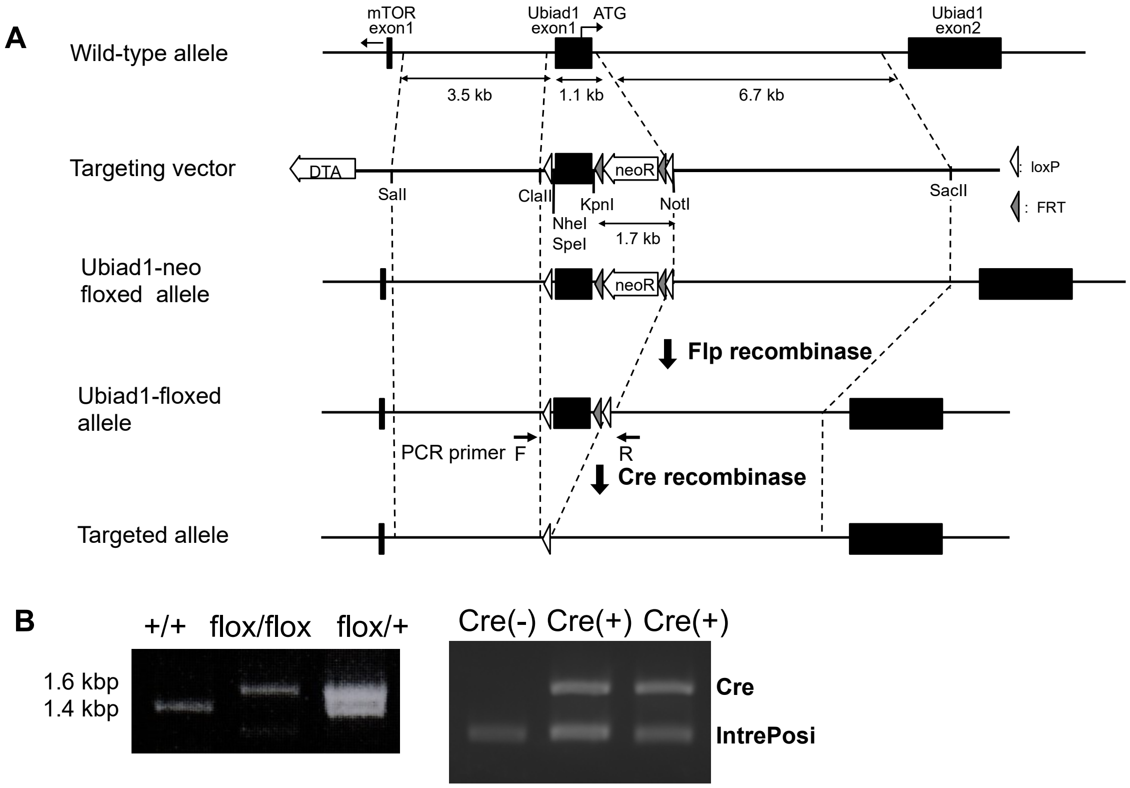

Figure 1. Generation of conditional *Ubiad1* knockout mice. (A) Schematic presentation of *Ubiad1* genome, targeting vector, and disrupted *Ubiad1* genome. (B) PCR genotyping of UBIAD1$^{+/+}$, *Ubiad1*$^{flox/+}$ and *Ubiad1*$^{flox/flox}$ fetus. PCR genotyping of tail DNA of UBIAD1$^{+/+}$, *Ubiad1*$^{flox/+}$ and *Ubiad1*$^{flox/flox}$ fetus. Lane 1, PCR bands of UBIAD1$^{+/+}$ fetus. Lane 2, PCR bands of CAG-Cre-ERT$^{+/-}$UBIAD1$^{fl/fl}$ fetus. Lane 3, PCR bands of CAG-Cre-ERT$^{+/-}$UBIAD1$^{fl/+}$ fetus.

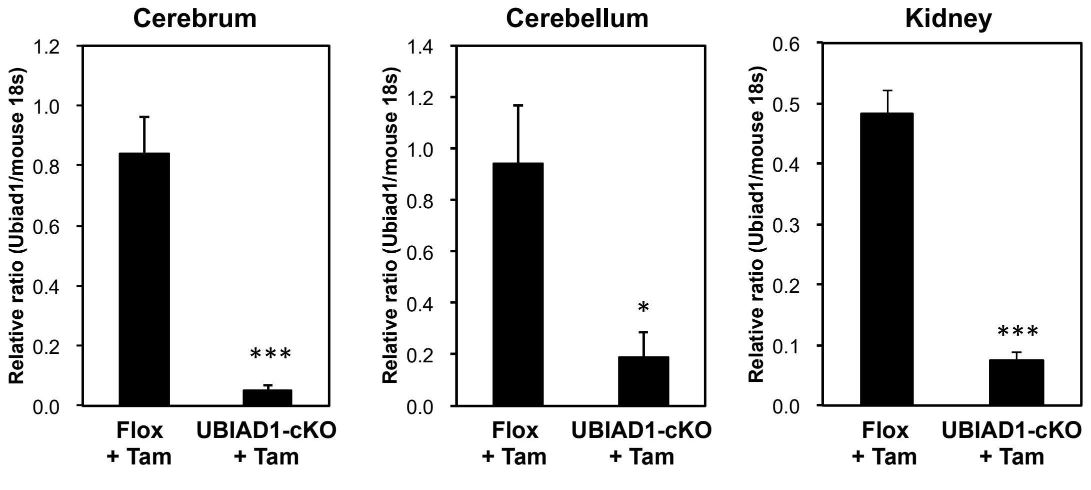

Figure S2. Ubiad1 mRNA expression in the cerebrum, cerebellum, and kidney of female Flox and UBIAD1-cKO mice at 40 days after administration of tamoxifen. * $p < 0.05$, *** $p < 0.001$ when compared with the corresponding values in the Flox + Tam mice.
